# Patient-centred consent in women’s health: does it really work in antenatal and intra-partum care?

**DOI:** 10.1186/s12884-022-04493-6

**Published:** 2022-02-25

**Authors:** Jacqueline Nicholls, Anna L David, Joseph Iskaros, Anne Lanceley

**Affiliations:** 1 EGA Institute for Women’s Health, Faculty of Population Health Sciences, University College London, Medical School Building, 74 Huntley Street, WC1E 6AU London, UK; 2 Elizabeth Garrett Anderson Wing, University College London Hospital NHS Foundation Trust, 25 Grafton Way, WC1E 6DB London, UK; 3 Research & Development, NIHR University College London Hospitals Biomedical Research Centre, 149 Tottenham Court Road, W1T 7DN London, UK

**Keywords:** Consent, Antenatal care, Intra-partum care, Healthcare professionals’ views, Choice, Women’s health

## Abstract

**Background:**

Legal and social changes mean that information sharing and consent in antenatal and intrapartum settings is contentious, poorly understood and uncertain for healthcare professionals. This study aimed to investigate healthcare professionals’ views and experiences of the consent process in antenatal and intrapartum care.

**Methods:**

Qualitative research performed in a large urban teaching hospital in London. Fifteen healthcare professionals (obstetricians and midwives) participated in semi-structured in-depth interviews. Data were collectively analysed to identify themes in the experiences of the consent process.

**Results:**

Three themes were identified: (1) Shared decision-making and shared responsibility –engaging women in dialogue is often difficult and, even when achieved, women are not always able or do not wish to share responsibility for decisions (2) Second-guessing women – assessing what is important to a woman is inherently difficult so healthcare professionals sometimes feel forced to anticipate a woman’s views (3) Challenging professional contexts – healthcare professionals are disquieted by consent practice in the Labour ward setting which is often at odds with legal and professional guidance.

**Conclusions:**

Results suggest that there is a mismatch between what is required of healthcare professionals to effect an antenatal or intrapartum consent process concordant with current legal and professional guidance and what can be achieved in practice. If consent, as currently articulated, is to remain the barometer for current practice, healthcare professionals need more support in ways of enabling women to make decisions which healthcare professionals feel confident are autonomous whatever the circumstances of the consultation.

## Background

Informed consent is an integral part of good healthcare practice in which autonomous patient-centred choice-making is central [1, 2]. In the United Kingdom, echoing a decision taken by the Australian Courts in the 1990’s [3], the decision in *Montgomery* and subsequent cases [5–9] reflects increasing legal endorsement of the importance of consultation dialogues which foster patient autonomy and so act as an ethical counter-balance to possibly unhelpful paternalistic or autocratic professional practices. In the landmark case of Montgomery [4] which concerned the failure of an obstetrician to inform a woman of the risk of shoulder dystocia associated with vaginal delivery the court stated that a doctor has a duty to ensure a patient is made aware of any material risks involved in treatment and of reasonable alternatives. What is material is determined both by reference to what a reasonable person in the patient’s situation might want to know and what the particular patient would be likely to attach significance to according to her concerns, beliefs and values. Underpinning the Court’s decision was the insistence that the dynamics of the healthcare professional-patient relationship should be characterised by respect for patient autonomy defined in the broadest of terms. Rather than relying on a classic model of autonomy in which a woman is conceived atomistically as an in-control moral agent who, as a rational recipient of adequately communicated complex risk information, is free to respond as she thinks fit, these terms point towards a recognition that women’s decision-making occurs within multiple relational and social contexts which may shape their decision-making [10–12]. While striking a further blow to medical paternalism the decision provoked fierce debate between those who felt information-sharing practice was radically changed in potentially uncertain ways and those who felt that the judgment merely restated the guidance set out in the General Medical Council’s Good Medical Practice [13]

 Determining what high quality patient consent looks like has become a key contemporary professional challenge [14]. Our aim was to explore the experiences of healthcare professionals (HCP) in relation to the challenge posed by antenatal and intrapartum consent consultations.

## Methods

This study is reported with reference to the consolidated criteria for reporting qualitative research (COREQ) [15]. A previously described interpretive qualitative methodology using in-depth interviews was used to understand how the consent process is viewed [16].

Study participants.

A convenience sample of HCP was recruited from an urban teaching hospital providing antenatal healthcare to approximately 6500 women annually. Eligible HCP were practicing obstetric doctors or midwives responsible for managing consultations involving consent for healthcare interventions such as caesarean section, cervical cerclage, instrumental delivery, episiotomy, and prenatal procedures. HCP were informed of the study via internal staff communications and were provided with an Information Sheet which detailed the researcher’s aims and interests in conducting the study. Participants were invited to ask questions prior to written informed consent.

 All procedures were in accordance with the Declaration of Helsinki and ethical approval was obtained from the UK Health Research Authority.

Data collection.

All semi-structured one-on-one interviews were conducted using a pilot-tested interview schedule (Table 1) by author JN, an associate professor and experienced female researcher with interests and research experience in women’s health and health law. Field notes were made immediately after each interview to contribute to analytic reflections and reflexive research considerations.

**Table 1:** Interview topic guide

**What do you think is the main purpose of the consent process?** (Prompts: How does it relate to your view of shared decision making? How do you approach the issue of consent with a patient? Is consent usually the responsibility of one member of the clinical team or does it involve several members? Who is usually responsible? What preparations do you make? What do you think the patient’s role is in the consent process?
**What issues and information are covered as part of the consent process?** (Prompts: What do you explain during the consent process? What do you think are the important things to address when discussing consent with a patient? What factors influence the information you give to a patient during the consent process. Do you discuss risk? How? Do you discuss benefit? How? What patient information sources do you use when seeking consent? Information sheets? Websites? Other?
**Are you aware of the** Montgomery **case?** (Prompts: What do you know about it? Has it influenced your practice? What difficulties, if any, have you experienced when seeking consent? Do you check a patient’s understanding when seeking consent? How do you assess whether a patient has understood?
How useful do you find the consent form? How long does it usually take you to seek consent?

### Data analysis

All audio-recorded interviews were anonymised and transcribed verbatim. To ensure validity and quality of the transcribed data the transcripts were double-checked against the original recordings. Data saturation was reached and demonstrated by the final interviews and was confirmed during initial coding.

Thematic analysis was used to analyse the interview transcripts. Following data familiarisation initial codes were generated in a systematic manner for the entire data set. Codes were collated into potential themes, where similar but separate codes were combined and refined. Transcripts were coded by JN using qualitative data analysis software [17]. To ensure consistency a random selection of 20% of the transcripts were coded independently by AL and differences were discussed until agreement was reached. Themes were reviewed, refined and organised into a final set of organising themes.

The researchers were all HCPs trained in good clinical practice with regard to research studies. AD and JI are practicing obstetricians. AL and JN are experienced women’s healthcare researchers.

## Results

Fifteen HCP (12 female (six obstetricians, six midwives); 3 male (two obstetricians, one midwife) participated, age range (25-59). Participants were not reimbursed for their participation. Interviews lasted 20–60 min. To ensure anonymity in this single site study, quotations are attributed by staff role and gender only. All transcripts revealed that HCP recognised the difficulties inherent in consent consultations. Three key overlapping themes were identified:

## Theme 1: Shared decision-making – shared responsibility?

 All participants were committed to shared decision-making but recognised it was sometimes challenging to engage women in dialogue. Concerns related to women who either did not want to receive much information or else wanted the HCP to decide for them about the proposed intervention. Included in the former group were concerns about women who were very passive - ‘just seem to be going along with whatever I say’.

I think they [women] need to be at the centre of it but it’s difficult because quite often they want to be more passive I suppose than that … I mean not necessarily in a bad way but … they do in some way feel they want us to tell them what they should do (HCP 10, female midwife).

I sometimes think they don’t really want to know, and I think sometimes we do just go with that which isn’t very good from a consent point of view but it’s just very difficult, so you just have to make sure you write it down (HCP 02, female midwife).

Conversely, some clinicians felt they were being asked to do the impossible – supporting women who wanted to make their own decisions provided that the responsibility lay firmly with the HCP if things went wrong.

it sometimes feels as though a woman wants to make all her own decisions, which is fine, but she doesn’t want to take any responsibility if it all goes pear-shaped’ (HCP 08, female obstetrician).

Allied to these were some concerns about women who were considered to be lacking the capability to decide.

there are women who are just undecided or who feel emotionally overwhelmed and sometimes they really just want you to make the decision for them … I’ve had patients who really struggle because they haven’t really got the ability, mentally speaking that is, to get their head round their situation … I think there are some women particularly on labour ward but not only there where at least at some points I feel uncomfortable about consenting them because on any normal understanding it would be very hard to say they are in a position to make an autonomous decision (HCP 13, female obstetrician).

it’s very interesting about capacity because you get women with some sort of obvious learning disability or psychiatric order and they can be quite tricky so you sometimes need to get the psychs in but also, those cases aside, the ones that also bother me are where an apparently ‘normal’ woman seems not to be taking on board what I’m saying either because she’s in pain or frightened or for some other reason and I sometimes think we don’t think about that enough when we consent them (HCP 06, female obstetrician).

the woman is panicking the partner is panicking and the doctors say just sign this so they just sign it and you can see they haven’t a clue what they’ve signed (HCP 03, female midwife).

Although some HCP felt their practice was unchanged following the Montgomery judgment many reported being more cautious when obtaining consent and more likely to accede to a woman’s wishes than prior to the judgment.

## Theme 2: Second-guessing women

All HCP expressed an intention to treat women as individuals and counsel them accordingly. Most also experienced difficulty in assessing what was important to an individual woman particularly within the confines of a time-limited consultation. They considered that making the sort of judgment endorsed by the Montgomery judgment required a degree of second-guessing of a woman’s beliefs and values.

someone’s situation may not be what it seems so I may not know what’s affecting their decision even though I think I do from a clinical point of view if that makes sense and that does really worry me because it feels as though I’m being asked to second-guess a woman if you see what I mean (HCP 14, female obstetrician).

I know that Montgomery tells us to think about a woman’s specific situation and obviously I try to do that but it’s really hard because as I said before I may not have met the woman before or even if I have, I may not know much about how she processes information, so it feels a bit impossible really (HCP 15, female obstetrician).

some people they’re very influenced by what is OK in their background, and for their ethnic group but obviously it’s quite hard to know really so it makes the whole consent thing a very grey area (HCP 04, male midwife).

Many HCP reported difficulty in striking a balance between fully disclosing all risks and avoiding unduly alarming a woman.

balancing the amount of information is not always easy … sometimes you have a finding and need to tell the patient what the options are, but you don’t know the patient very well, so you don’t know how she’s going to be able to process all the information you’re going to give (HCP 05, male obstetrician).

[post-Montgomery] I‘m more careful about labouring the risks more and making sure I mention everything because I do think it’s really hard to know what matters to someone - you know, their idea of risk depends on their belief system so you’re sometimes trying to really form a picture of what you think they’re like in a very short time (HCP 13, female obstetrician).

Allied to this was the difficulty in gauging a woman’s attitude to risk in general as well as their stance on risks.

it’s difficult trying to work out how risk averse they are because we get women who jump at 1 in 200 and are really upset about it and then we get women where the risk is 1 in 7 and they’re like ‘oh it’s fine don’t worry about it’ so it’s gauging how they perceive risk (HCP 01, female midwife).

These difficulties were intensified when counselling women with poor language skills and in these circumstances HCP were frequently left feeling uneasy that the requirements of valid consent had been met.

if a woman doesn’t speak any English so you’re totally reliant on Language line or quite often on family members and that’s tricky in generally understanding what matters to a woman which is difficult enough anyway let alone in terms of communicating risk … I’ve had a few women when I’ve had real doubts about whether they really get it, you know they just nod to everything and then sign the form when they’re prompted to do which obviously isn’t consent in the way we normally understand (HCP 08, female obstetrician).

## Theme 3: Challenging professional contexts - the Labour Ward

Concerns relating to consent obtained for interventions in the Labour ward context such as emergency caesarean section and instrumental delivery were raised by many HCP.

it’s very difficult because consent on the labour ward is not black and white, actually category 1 emergencies and caesarean section are in a way very easy, it’s either we get your baby out or there’s going to be damage to your baby but there are some areas where it’s not that black and white and that’s quite tricky (HCP 06, female obstetrician).

on the Labour ward (pulls a face) it’s really, really not good – I mean the whole circumstances often, not always, but often, are just so rushed and not compatible with doing proper consent so I really hate that (HCP 10, female midwife).

They reported experiences in which the process of obtaining consent had been absent, cursory, or poorly-informed with negligible engagement from the pregnant woman concerned who might be suffering pain or fatigue.

I really think that consent in labour is really dodgy for lots of things, I mean take wide perineal suturing, by that stage most women are really beyond caring, and you know I’ve seen women subsequently who said they had no idea what was going on so yes, I think to say consent is dodgy is an under-statement (HCP 09, female obstetrician).

there’s quite a lot of times when it’s not [an emergency] but the woman gets consented in really what is a pretty cursory way and especially if she’s in pain or just had enough it really isn’t anything like proper consent …. it’s often the case with women who have got it firmly in their minds that they want a natural birth so you’re trying to persuade them I suppose to think about having a section and time’s pressing on so it’s quite easy if I’m honest to consent them in a fairly perfunctory way and I do think sometimes that’s a bit dodgy… it goes against everything you normally try to do in terms of professional practice. I mean obviously it is still their decision, but I think it’s in a very notional way because of the situation and I sometimes wonder if we not exactly abuse but take advantage of the situation (HCP 15, female obstetrician).

Many HCP had experienced fast moving situations where they felt lawful consent according to the Montgomery judgment had not been obtained and this left them feeling stressed and uneasy.

[Labour Ward]… it’s moving so fast and I am very conscious of all the things you normally think of with consent tend to go out of the window, you know they’re in pain it’s all a bit tense and so on I mean it’s probably one of the things I feel most uncomfortable about in all of my practice … you know you’re not in line professionally with everything that’s been drummed into you from an early stage and it’s difficult to do anything about it (HCP 14, female obstetrician).

you basically end up with what you might call kneejerk consent rather than anything that’s really appropriate, so you’re conflicted because you’re wanting to offer patient-centred care but it’s not (HCP 13, female obstetrician).

## Discussion

Healthcare professionals in this study recognised that truly autonomous shared decision-making can be difficult to achieve both antenatally and intrapartum across the spectrum of women ranging from those who appear overly compliant or disinterested to those who want to decide but do not wish to take responsibility for their decision. The latter represents a challenge which highlights the fine professional boundary between providing information and proffering advice and may indicate a need for greater clarification of this distinction.

Despite the psychological challenges accompanying pregnancy we were surprised to find that many HCP reported concerns about the decision-making capacities of some women, often, though not exclusively, when women were being asked for consent in the acute Labour ward setting. Similar concerns have been both voiced [18, 19] and refuted [20] in the pre-Montgomery era in but more recently Singh [21] et al. found incapacity in obstetric emergency procedures to be relatively common. Interestingly, a common misapprehension is that the assessment of capacity should be performed by a psychiatrist whereas the MCA [22] states that it is the clinician responsible for care who is responsible for carrying out an assessment. Limited previous work [23] indicates that HCPs have a poor understanding of the MCA and its use in practice and we suggest that further investigation of the support offered to HCP facing these concerns may be justified.

HCP were divided in relation to whether the Montgomery judgment had changed their practice, but it seems that rather than a seismic change in practice, what judgments such as Montgomery and sequelae cases [24–27] have done is highlighted the uncertainty inherent in the complexity of clinical consultations and embroidered it with the additional uncertainty of the materiality test. Professional guidance [28] has long recognised the need for HCP to take full account of factors relevant to their patients’ individual decision-making. Cases such as Montgomery re-emphasise that consent is not a mechanical process in which the materiality of a risk can be determined by its numerical likelihood or similar evidence. Whilst such approaches are meritorious within the frame of evidence-based healthcare they do not take into account the increasingly acknowledged need for a consultation dialogue framed by an appreciation of a woman’s values. Many of the HCP in our study appeared to recognise what might be seen as an imbalance between evidence and values-based healthcare [29] and we found real concern among HCP about the ‘correct’ way of counselling women and ascertaining their values, beliefs and preferences. Making these sorts of assessments is undoubtedly challenging [30, 31] but aligns with increasing recognition across the globe that adopting a woman-centred philosophy and human-rights based approach as endorsed by the World Health Organisation is central to giving women authentic involvement in decision-making [33]. Hence we suggest, meeting these challenges is necessary to support high quality, lawful, professional consent practice and requires the development of better tools to embed value/belief/preference assessments in clinical consultations.

Our findings confirm previous suggestions [33] that consent in the LW setting is sub-standard even in non-emergency circumstances. Echoing the findings of others [34] recalling the effects of pain and fatigue on labouring women caused many HCP to doubt if some women had the mental capacity to make a decision at the point in time when their consent was sought. Even when capacity was present clinicians had reservations about whether ‘proper’ consent had been obtained. Perhaps HCP should begin the consent process by formally discussing the risks of potential interventions such as caesarean section, instrumental delivery and episiotomy well ahead of labour and delivery with the process being completed if, and when, such interventions become necessary. Clearly, in order to mitigate concerns about unwarranted intrusion on women antenatally and intrapartum, such approaches to consent would need to be carefully and sensitively designed. Nonetheless whether consent requirements as currently formulated are really supportive of good clinical care and, if not, how they can be implemented effectively clearly warrants further investigation.

This small study is, we believe, the first published assessment of the experiences of both practicing midwives and obstetricians post-Montgomery. Despite being a single site study it provides a useful opportunity to explore HCP perspectives at an institution representative of other large UK teaching hospitals both in terms of staff and numbers of women attending for care. However, although data saturation was evidenced and many key issues were revealed, professional practice in general and consent in particular is multi-faceted and we may have failed to capture some nuances of particular challenges and experiences.

## Conclusions

Legal & professional guidance on patient consent sets a high bar for healthcare professionals. Yet what is required of healthcare professionals is mismatched with what can be achieved in practice. If consent, as currently formulated, is to remain the yardstick for practice, healthcare professionals need more support in ways of enabling women to make decisions which they can feel confident are truly autonomous whatever the circumstances of the consultation.

## Data Availability

The data were collected on a confidential basis so fully anonymised datasets are available from the corresponding author on reasonable request from authenticated researchers. The study was conducted by HEFCE funded researchers.

## Abbreviations

HCP: Healthcare professionals

## References

[1] Barry MJ, Edgman-Levitan S. Shared decision making–pinnacle of patient-centred care. N Engl J Med. 2012;366 (9):T 780–1. 10.1056/NEJMp1109283 22375967

[2] Beermat JL, Peterson LM. Patient-centred informed consent in surgical practice. Arch Surg. 2006;141 (1):86–92. 10.1001/archsurg.141.1.86 16415417

[3] Rogers v Whitaker 1992 175 CLR 479.

[4] Montgomery v Lanarkshire Health Board. 2015; UKSC 11 UK Supreme Court.

[5] FM (by his father and litigation friend GM) v Ipswich Hospital NHS Foundation Trust. 2015;EWCH 775.

[6] Webster v Burton Hospital NHS Foundation Trust 2017; EWCA Civ 62.

[7] Thefault v Johnston 2017 EWHC 497 (QB).

[8] *Diamond v Royal Devon* & Exeter NHS Foundation Trust [2019] EWCA Civ 585. 10.1093/medlaw/fwz020 31347652

[9] Duce v Worcestershire Acute Hospitals NHS Trust 2018 EWCA Civ 1307.

[10] Wolpe P, DeVries R, Subedi J (1998). The Triumph of Autonomy in American Bioethics: a sociological view. Bioethics and society: constructing the ethical enterprise.

[11] Mackenzie C, Stoljar N. Autonomy Refigured: In Mackenzie C, Stoljar N (eds) Relational autonomy; feminist perspectives on autonomy, agency and the self. Oxford: OUP; 2000. p. 3–34.

[12] Donchin A. Autonomy and Interdependence: In Mackenzie C, Stoljar N (eds) Relational autonomy; feminist perspectives on autonomy, agency and the self. Oxford: OUP; 2000. p. 236–58.

[13] General Medical Council. 2020 Consent: patients and doctors making decisions together. Manchester.

[14] Devaney S, Purshouse C, Cave E, Heywood R, Miola J, Reinach N. The far-reaching implications of Montgomery for risk disclosure in practice. J Pat Saf Risk Manag. 2018;24(1):25–9.

[15] Tong A, Sainsbury P, Craig C. Consolidated criteria for reporting qualitative research (COREQ): a 32 item checklist for interviews and focus groups. Int. J. Qual. Health Care. 2007;19(6 ):349–57. 10.1093/intqhc/mzm042 17872937

[16] Nicholls J, David, A, Iskaros J, Lanceley A. Consent in pregnancy: A qualitative study of the views and experiences of women and their healthcare professionals. Europ. J Obst Gynecol. Reprod. Biol. 2019;238:132–7. 10.1016/j.ejogrb.2019.05.008 31129561

[17] QSR NVivo 1010 (QSR International, Cambridge, MA).

[18] Saunders TA, Stein DJ, Dilger JP. Informed consent for labour epidurals: A survey of Society for Obstetric Anaesthesia and Perinatology anesthesiologists from the United States. Intnl J Obstet Anaest. 2006;15(2): 98–103. 10.1016/j.ijoa.2005.08.002 16434182

[19] Goldberg HB, Shorten A. Differences between patient and provider perceptions of informed decision making about epidural analgesia use during childbirth. J Perinatal Education. 2014; 23(2):104–12. 10.1891/1058-1243.23.2.104 PMC3976641 24839385

[20] Stohl H (2018). Childbirth Is Not a Medical Emergency: Maternal Right to Informed Consent throughout Labor and Delivery. J Leg Med.

[21] Singh N, Lepping P, Whitaker R, Masood B, Joshi S, Banfield P (2021). Incapacity in childbirth – Rare or common?. Eur J Obstet J Obst. & Gynecol. Reprod. Biol. X.

[22] Department of Health. Mental Capacity Act. London: HMSO; 2005.

[23] Penn D, Lanceley A, Petrie A, Nicholls J (2021). Mental capacity assessment: a descriptive, cross-sectional study of what doctors think, know and do. J Med Ethics.

[24] Spencer v Hillingdon Hospital NHS trust [2015] EWHC 1058 (QB).

[25] A v East Kent Hospitals University NHS Foundation Trust [2015] EWHC 1038 (QB).

[26] Gallardo v Imperial College Healthcare NHS trust [2017] EWHC 3147.

[27] Tasmin v Barts Health NHS Trust [2015] EWHC 3135 (QB).

[28] General Medical Council. 2008; Consent: patients and doctors making decisions together. Manchester.

[29] Fulford KW. Values-based practice: a new partner to evidence-based practice and a first for psychiatry? Mens Sana Monogr. 2008;6 (1):10–21. 10.4103/0973-1229.40565. 10.4103/0973-1229.40565 PMC3190543 22013346

[30] Mangin D, Stephen G, Bismah V, Risdon C. Making patient values visible in healthcare: a systematic review of tools to assess patient treatment priorities and preferences in the context of multimorbidity. BMJ Open. 2016; 6(6): e010903. 10.1136/bmjopen-2015-010903. 10.1136/bmjopen-2015-010903 PMC4908882 27288377

[31] Soekhai V, Whichello C, Levitan B, Eldwijk J, Pinto C, Donkers B (2019). Methods for exploring and eliciting patient preferences in the medical product lifecycle: a literature review. Drug Discov. Today.

[32] WHO recommendations (2018). intrapartum care for a positive childbirth experience.

[33] Kennedy S, Lanceley A, Whitten M, Kelly C, Nicholls J. Consent on the Labour ward: a qualitative study of healthcare professionals’ views and experiences. Eur J Obstet J Obst Gynecol. Reprod. Biol. 2021;264:150–4. 10.1016/j.ejogrb.2021.07.003 34303075

[34] Smith M, Levy K, Yudin M. Informed Consent During Labour: Patient and Physician Perspectives. J Obstet Gynaecol Can. 2018;40(5):614–7. 10.1016/j.jogc.2017.12.013 29731207

